# The impact of implementing a hospital electronic prescribing and administration system on clinical pharmacists’ activities - a mixed methods study

**DOI:** 10.1186/s12913-019-3986-4

**Published:** 2019-03-12

**Authors:** Monsey McLeod, Georgios Dimitrios Karampatakis, Lore Heyligen, Ann McGinley, Bryony Dean Franklin

**Affiliations:** 1 Centre for Medication Safety and Service Quality, Imperial College Healthcare NHS Trust, Charing Cross Hospital, Fulham Palace Road, London, W6 8RF UK; 2 School of Pharmacy, University of Reading, Whiteknights Campus, PO Box 226, Reading, RG6 6AP UK; 3 Faculty of Pharmaceutical Sciences, Catholic University of Leuven, Campus Gasthuisberg, O&N II, Herestraat 49 bus 420, 3000 Leuven, Belgium; 4 Pharmacy Department, Imperial College Healthcare NHS Trust, Charing Cross Hospital, Fulham Palace Road, London, W6 8RF UK; 5 Research Department of Practice and Policy, UCL School of Pharmacy, Brunswick Square, London, WC1N 1AX UK

**Keywords:** Electronic prescribing, Electronic prescribing and medication administration system, Computerised physician order entry (CPOE), Pharmacist, Patient safety, Medication safety, Mixed methods, Work-sampling, Workflow, Interview

## Abstract

**Background:**

The increasing adoption of hospital electronic prescribing and medication administration (ePA) systems has driven a wealth of research around the impact on patient safety. Yet relatively little research has sought to understand the effects on staff, particularly pharmacists. We aimed to investigate the effects of ePA on pharmacists’ activities, including interactions with patients and health professionals, and their perceptions of medication safety risks.

**Methods:**

A mixed methods study comprising quantitative direct observations of ward pharmacists before and after implementation of ePA in an English hospital, and semi-structured interviews post-ePA. Quantitative data comprised multi-dimensional work activity sampling to establish the proportion of time ward pharmacists spent on different tasks, with whom and where. These data were extrapolated to estimate task duration. Qualitative interviews with pharmacists explored perceived impact on (i) ward activities, (ii) interactions with patients and different health professionals, (iii) locations where tasks were carried out, and (iv) medication errors.

**Results:**

Observations totalled 116 h and 50 min. Task duration analysis suggested screening inpatient medication increased by 16 mins per 10 patients reviewed (*p* = 0.002), and searching for paper drug charts or computer decreased by 2 mins per 10 patients reviewed (*p* = 0.001). Pharmacists mainly worked alone (58% of time pre- and 65% post-ePA, *p* = 0.17), with patient interactions reducing from 5 to 2% of time (*p* = 0.03). Seven main themes were identified from the interviews, underpinned by a core explanatory concept around the enhanced and shifting role of the ward pharmacist post-ePA. Pharmacists perceived there to be a number of valuable safety features with ePA. However, paradoxically, some of these may have also inadvertently contributed to medication errors.

**Conclusion:**

This study provides quantitative and qualitative insights into the effects of implementing ePA on ward pharmacists’ activities. Some tasks took longer while others reduced, and pharmacists may spend less time with patients with ePA. Pharmacists valued a number of safety features associated with ePA but also perceived an overall increase in medication risk. Pharmacy staff demonstrated a degree of resilience to ensure ‘business as usual’ by enhancing and adapting their role.

**Electronic supplementary material:**

The online version of this article (10.1186/s12913-019-3986-4) contains supplementary material, which is available to authorized users.

## Background

The uptake of hospital inpatient electronic prescribing and medication administration (ePA) systems is increasing, with benefits cited as reduced adverse drug events, medication errors, length of stay, and healthcare costs, and more accurate communication among healthcare professionals [1–5]. However, other evidence challenges the extent of such benefits [6, 7]. The adoption of a complex intervention such as ePA will bring about considerable organizational change, especially in the daily work of frontline staff [8–10]. Not surprisingly, there are cases of unsuccessful ePA implementations with the main reason cited as users’ resistance [11, 12]. However, relatively little research has focused on the impact of ePA on the people and processes required to realise the improvements in patient care.

In England, pharmacists provide an important ward-based clinical service to inpatients and support to ward staff [13]. The three English studies in this area provide a mixed picture of the impact of ePA on ward pharmacists. Some suggest pharmacists spend less time with patients [7, 14] and doctors post-ePA [7, 15], and require increased time for ward activities [7, 15]. Another suggests no change in time with patients [15] and a reduction in time on specific tasks such as ordering medications, searching for drug charts and checking patients’ own drugs [15]. An Australian study suggests pharmacists spend less time with patients as drug charts were no longer at their bedside, more time working alone, were interrupted less often and spent less time reviewing medications [16]. However, these studies were conducted some time ago in hospitals where ePA was implemented on few wards, and mobile computing devices were not always available or were too cumbersome to use at patients’ bedsides. Hospital-wide ePA may have a different impact on pharmacists’ activities and medication safety, as suggested by more recent US studies [17, 18]. Understanding the wider effects in an English hospital setting is particularly pertinent as significant National Health Service (NHS) funds are being used to incentivise greater uptake of ePA [19]. Greater understanding is also essential to aid workforce planning and to influence staff acceptance and use of ePA to improve patient safety. The aim of this study was to investigate the impact of implementing a hospital-wide ePA system on ward pharmacists’ activities, including their interactions with patients and health professionals, and their perceptions of medication safety risks.

## Methods

### Study design

This was an uncontrolled before-and-after study using an explanatory sequential mixed methods design [20]. This design enables quantitative results to be explained using qualitative methods and was chosen to provide greater understanding of the effects of ePA on ward pharmacy practice. Quantitative activity sampling of ward pharmacists’ work before and after implementation of a hospital-wide ePA system was therefore followed by semi-structured qualitative interviews.

### Setting

We studied an acute admissions ward and a medicine-for-the-elderly ward in a large English NHS teaching hospital. These wards were purposively chosen to represent differences in patient length of stay, ward staff working practices, and pharmacy services. Typical UK hospital ward pharmacist activities were carried out on both wards, including taking medication histories, reconciling medications on admission, reviewing medication orders for clinical appropriateness, ordering medications, reviewing medications for discharge, patient counselling and some ward-based dispensing. On the acute admissions ward, pharmacy staff carried out these activities seven days a week as part of a ward-based service. This also included attendance at each of the two consultant-led ward rounds Mondays to Fridays, and one each Saturday and Sunday. In contrast, on the medicine-for-the-elderly ward, a single pharmacist provided a two hour ward visit Monday to Friday and attended one consultant-led ward round each week. The ePA system, a component of a commercial electronic health record system already in use at the hospital, was implemented on both study wards in March 2016 as part of the second phase of hospital-wide roll-out. Clinical decision support within the ePA system mainly comprised tools such documentation templates, condition-specific order sets, and medication-specific review reminders. Drug allergy alerts were in operation, but other computerised alerts such as drug-drug interactions were not activated. There was no formal change in ward pharmacy resources provided post-ePA.

### Recruitment and consent

All eight pharmacists covering both study wards were invited to participate in the observations and interviews. Those willing to take part provided written consent and mutually convenient time(s) and day(s) for observations and interviews were agreed.

### Quantitative work activity sampling

Direct observations of pharmacists’ ward visits were planned for every 3–4 weekdays on each ward between January 2016 (approximately 1.5 months pre-ePA) and June 2016 (3 months post-ePA). One observation session typically lasted around two hours. Observations were scheduled to take place at two different times (after the morning ward round and late afternoon) on the acute admissions ward to capture potential variation in ward-based activities as it was impractical to observe the pharmacist for their entire eight-hour shift. As pharmacists on the medicine-for-the-elderly ward were not ward-based all day, observations were scheduled in the morning for the duration of their ward visit and they were asked to document any ward-related activities undertaken outside the ward visit using pre-piloted data collection forms on the days observed. Additionally, the presence of any other pharmacy staff on the ward was recorded.

A standard random interval three-dimensional work sampling approach [21] was used by LH to collect data on (i) different types of tasks carried out, (ii) with whom, and (iii) where. Briefly, a random signal generator (JD-7, Divilbliss Electronics, Champaign, Illinois) was set to produce 32 silent vibration alerts (‘activity samples’) per hour with random intervals between successive alerts. The researcher recorded data on pre-piloted forms each time an alert was produced e.g. a two-hour observation would be expected to generate 64 alerts and therefore 64 activity samples would be documented. This can be used to provide an estimate of the proportion of overall time spent on the different tasks [22]. The same researcher (LH) collected data both pre- and post-ePA and carried out a series of pilot observations on the study wards to become familiar with the data collection tool, and to allow ward staff to become familiar with being observed, before starting data collection. An existing task list [23] was expanded from 11 to 20 tasks (Additional file 1) by further subdividing existing categories to provide greater granularity and facilitate identification of task changes that were potentially more ePA-specific. Each task was also assigned by one researcher (MM) as being either value-added or non-value-added from a patient’s perspective, based on Lean methodology, to facilitate identification of potentially ‘wasteful’ or non-value-added steps [24]. This classification was verified separately by a second member of the research team (BDF). Contextual data such as pharmacists’ post-qualification years of experience, number of patients reviewed, and observation start and stop times were also recorded.

### Qualitative semi-structured interviews

One-to-one semi-structured interviews were carried out by GK in May 2016 (approximately seven weeks post-ePA) using a pre-piloted topic guide Additional file 2. Pharmacists were asked questions around their perceptions of the impact of ePA on (i) their ward activities, (ii) interactions with patients and different health professionals, (iii) locations where tasks were carried out, and (iv) medication errors. Interviews were audio recorded and transcribed verbatim.

### Data analysis

Quantitative analysis focused on the change in estimated time spent on value-added and non-value-added tasks on both wards. Estimates of time were based on ten patients reviewed to provide context with respect to implications for pharmacy services and to take into account any changes in numbers of patients reviewed. The percentage of time spent on each task overall was also calculated to provide an overview of how pharmacists spent their time. As this was an exploratory study, an a priori sample size was not calculated. We used the Bonferroni correction for multiple testing and adopted a *p*-value of 0.0028 for assessing whether differences in task duration were statistically significant. We also compared median time per patient reviewed. Mann-Whitney U tests (for non-parametric data) and unpaired t-tests (for parametric data), with a two-tailed significance of 0.05, were used to explore potential differences between mean percentage of time spent on different tasks per observation, and mean percentage of time per observation with patients, different health professionals, and locations where tasks were carried out pre- and post-ePA.

Qualitative data were analysed using a deductive thematic approach with initial themes generated based on study objectives [25]. One researcher (GK) reviewed all interview recordings and transcripts to maximise familiarity with the data prior to coding and adjusted the coding frame accordingly. Each transcript was coded manually on paper. A second researcher (MM) reviewed the coding and identified high level themes that were later refined with the rest of the research team before being finalised.

Findings from the quantitative and qualitative data analyses were compared to explore areas of agreement and disagreement between measured and perceived impact of ePA on pharmacists’ activities. The modified Good Reporting of Mixed Methods Study guideline for pharmacy practice was used to support transparent reporting [26].

## Results

Seven of the eight pharmacists were observed, over a total of 116 h and 50 min (Table 1). The median time per patient reviewed was 17 min pre- and 22 min post-ePA; the difference was not statistically significant (*p* = 0.16). Six of the seven pharmacists covered both the admissions and medicine-for-the-elderly wards during the observation period; these six pharmacists also participated in the interviews (experience 1.5 to 6.5 years). Additional pharmacy support from at least one other team member was available for some or all of the time observed on the acute admissions ward (84% of 44 observations) and were mainly provided by a pharmacy technician (73% of observations). An additional pharmacist was available pre- (39% of observations) and post-ePA (62% of observations), and pre-registration trainee support (pre-ePA 28% and post-ePA 0% of observations) on the acute admissions ward. Additional ward-related activities carried outside of the ward visit by pharmacists on the medicine-for-the-elderly ward were reported for only one day pre-ePA and three days post-ePA; these data were therefore excluded from analysis.

**Table 1:** Overview of observations

	Acute admissions			Medicine for the Elderly			Both wards combined		
	Pre-ePA	Post-ePA	*p*-value	Pre-ePA	Post-ePA	*p*-value	Pre-ePA	Post-ePA	*p*-value
Number of observations	18	26	–	6	13	–	24	39	–
Total observation time (h:min)	33:21	47:42	–	10:09	25:38	–	43:30	73:20	–
Mean duration of observation (h:min)	1:51 (SD 14 mins)	1:50 (SD 14 mins)	0.80	1:42 (SD 28 mins)	1:58 (SD 16 mins)	0.11	1:49 (SD 18 mins)	1:53 (SD 15 mins)	0.34
Median number of patients reviewed per observation	6 (range 2–10)	5 (range 1–16)	0.18	18 (range 3–22)	9 (range 3–22)	0.48	6 (range 2–22)	5 (range 1–22)	0.36
Number of activity samples	990	1424	–	297	752	–	1287	2176	–
Median time per patient reviewed (mins, 95% CI)	19 (15–24)	21 (20–30)	0.14	7 (4–22)	14 (5–24)	0.20	17 (15–22)	22 (17–24)	0.16

Abbreviations: *CI* confidence interval, *ePA* electronic prescribing and medication administration system, *SD* standard deviation

### Quantitative work sampling

Overall task analysis indicates that pharmacists spent most time on: professional communication (relating to patient care but unrelated to medication, 15.9% of time pre-ePA and 15.1% post-ePA, *p* = 0.62); screening inpatient medication (11.6% pre- and 17.4% post-ePA, *p* = 0.20); and screening discharge medication (11.0% pre- and 6.2% post-ePA, *p* = 0.45) Additional file 3. There was no statistically significant difference in the percentage of time spent on any of the individual tasks pre- and post-ePA except for taking a drug history (6.5% pre- and 13.2% post-ePA, *p* = 0.02).

#### Duration of value-added and non-value-added tasks

**Fig. 1:**
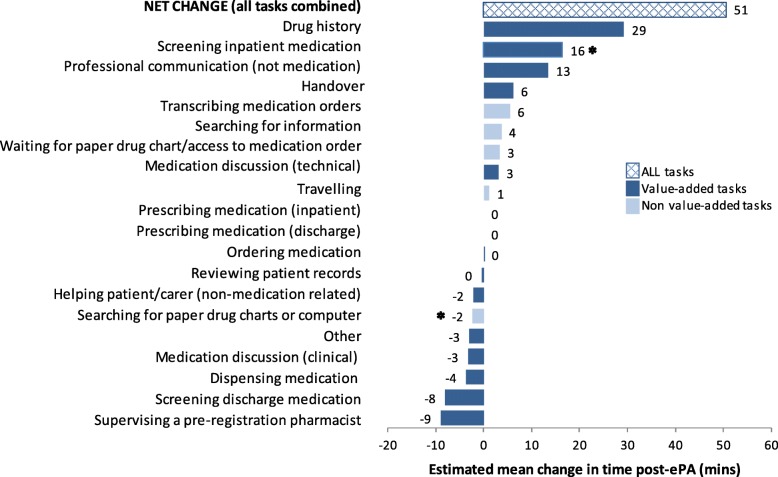
Estimated mean change in time per 10 patients reviewed by pharmacist post electronic prescribing and administration (ePA). *statistical significance based on Bonferroni *p*-value <0.0028 (unpaired t-test). ‘Other’: casual conversation, scheduled break, waiting for equipment to become available, researcher-related activity, searching for equipment, auditing, handwashing, and unknown tasks

There was no statistically significant difference in the total time spent on all tasks combined (value-added and non-value-added) per 10 patients reviewed post-ePA (Fig. 1). Only two specific tasks were associated with a significant change post-ePA: screening inpatient medication increased by 16 mins per 10 patients reviewed (*p* = 0.002), and searching for drug charts decreased by 2 mins per 10 patients reviewed (*p* = 0.001).

#### Interactions with patients/visitors and other health care professionals

**Fig. 2:**
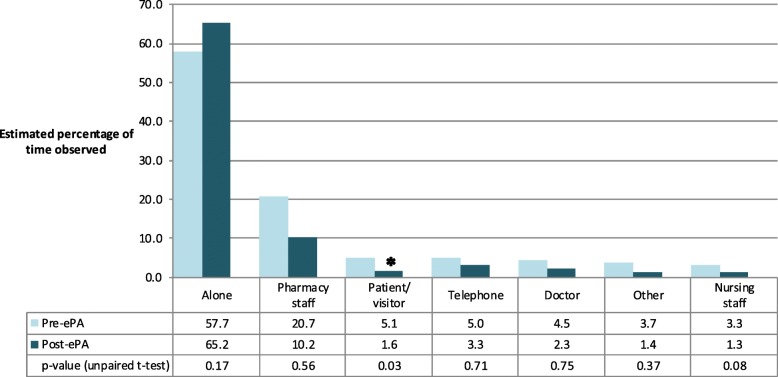
Estimated percentage of time observed working alone or interacting with others by pharmacist. Estimations were based on 1346 activity samples pre-electronic prescribing and administration (ePA) and 2176 post-ePA. Total exceeds 100% as pharmacists sometimes interacted with more than one other individual. * denotes *p*-value <0.05 (unpaired t-test)

Pharmacists spent the majority of their time working solo (Fig. 2) both pre- and post-ePA (60 and 65% of time respectively, with the difference not statistically significant). However, the percentage of time spent interacting with patients and/or their visitors was significantly less post-ePA (*p* = 0.03). Interactions with “other persons” included: ward clerk, occupational therapist, researcher, and information technology support staff.

#### Locations of work on the ward

**Fig. 3:**
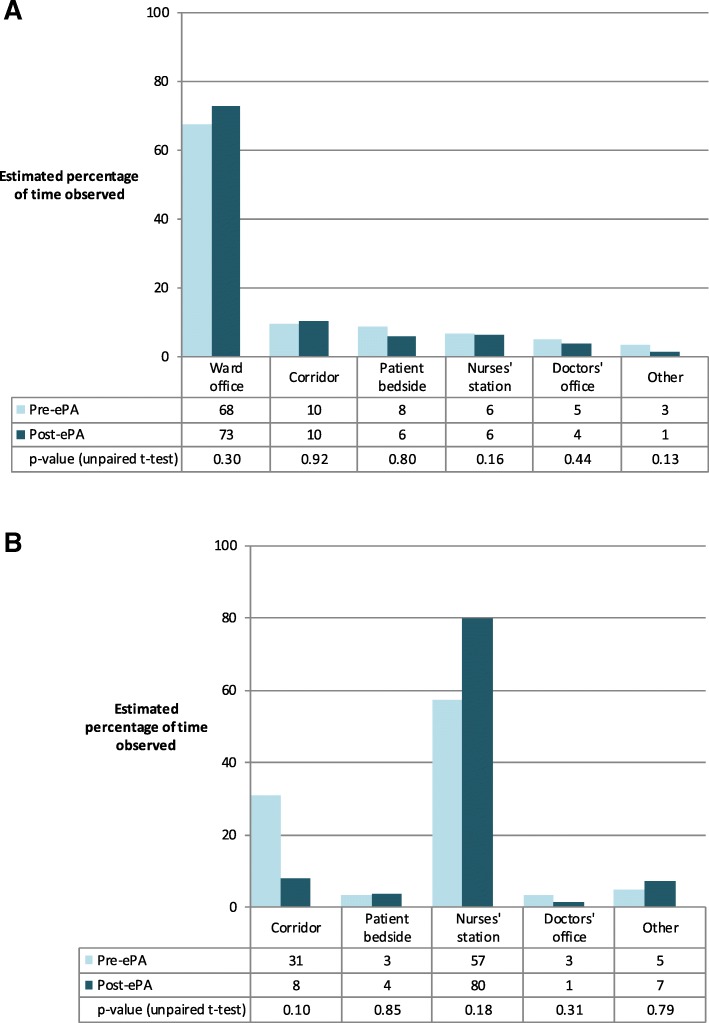
Estimated percentage of time observed working in different locations by pharmacist: (**a**) acute admissions, (**b**) medicine-for-the-elderly ward**.** Abbreviation: ePA, electronic prescribing and administration

On the acute admissions ward, pharmacists spent the majority of time observed working in the ward office, which changed little post-ePA (Fig. 3A). In comparison, pharmacists on the medicine-for-the-elderly ward appeared to shift from working in the corridor (where health records trolley and paper drug charts were sometimes located) to the nurses’ station (Fig. 3B); however, there was no statistically significant difference pre- and post-ePA. ‘Other’ locations for both wards were: medication storage room, patient bay (but not at bedside), visiting room, toilet, kitchen, and stairs.

### Interview findings

Overall, we identified seven main themes from the interview transcripts: (i) more efficient and effective screening of medications post-ePA, (ii) more time-consuming daily work, (iii) fewer patient interactions and a less natural communication mode, (iv) more interactions with other health professionals but less face-to-face communication, (v) discussions with other health professionals and patients are more focused on problem solving, (vi) patients valued over medical records as a reliable source of medication history, and (vii) higher prevalence of medication errors. These were interlinked by a core concept: the enhanced and shifting role of the ward pharmacist post-ePA. We present this concept below as the primary finding from integration of quantitative and qualitative data. Selected verbatim quotes are included for illustrative purposes.

#### The enhanced and shifting role of pharmacists post-ePA

A principal aspect of a ward pharmacist’s responsibilities is to clinically screen patients’ medications, ensuring accuracy as well as appropriateness and practicality for use. While this remains the case, interviews with pharmacists post-ePA suggest that they were also taking on more of a transcribing role by ordering medication based on a doctor’s verbal request plus an ePA support role for ward staff in general. Pharmacists reported being approached by doctors and other ward staff more frequently to make prescription changes on the ePA system, for advice on how to use the ePA system, and for troubleshooting.

*“Our role has changed a lot more to be a person who updates the actual drug chart and updates the actual prescriptions. Because it is a new system the junior doctors, I think rely a lot more heavily on the fact that we can PC [amend on behalf of a “prescriber contacted”] amendments or changes, or additions. ………. now just on ward round alone, the drug chart screen is only really looked at by us [pharmacists]”.* (Pharmacist 1; 4 years’ experience)

*“Yeah, technical issues, …….. definitely I’ve got more questions from nurses about what things mean on [the ePA system], or can I help them find something. Or if they’ve signed for something accidentally, how do they reverse it and things like that. And then from a doctors’ point of view, I suppose just feeding back things that they can do differently”.* (Pharmacist 2; 1.5 years’ experience)

Despite their greater transcribing and additional technical support roles, pharmacists themselves strived to continue to provide a ‘typical’ clinical pharmacy service and recognised many advantages and disadvantages of ePA. Pharmacists believed that the ePA system enabled more efficient screening of drug charts and a greater propensity to make meaningful clinical contributions for optimising a patient’s medications. Specific features of the ePA system, including a pharmacy medicines management page, were highlighted as helpful for prioritising patients at each visit. However, pharmacists perceived some common routine tasks, such as taking a medication history, to be more time-consuming.

*“….there’s a vast amount of information available now when I’m seeing those patients for a first time on ward round ….. that wasn’t available to me pre [ePA] … [the ePA system] allows everything to kind of be gathered into one… I find myself being able to screen a drug chart clinically with a lot more information on a ward round”* (Pharmacist 1, 4 years’ experience)

*“The time I think I spend putting information into [the ePA system] takes a lot longer post implementation. It takes a lot longer for me to do a drug history, do a meds rec’ [medicines reconciliation] and finish [with] a patient before I’m happy to move onto another one”.* (Pharmacist 1, 4 years’ experience)

Pharmacists who believed ePA facilitated clinical screening attributed this to having more convenient access to patient records that allowed them to review medications, laboratory results and medical notes concurrently. However, paradoxically, this meant pharmacists would sometimes spend more time reviewing a patient’s medications as they were reviewing the medical notes and laboratory results more frequently. Given that pharmacists reported little change in the way they prioritised their ward activities (i.e. reviewing new patients and those due to be discharged first), the consequence was that pharmacists carried out fewer patient reviews post-ePA for those who were not new or due to be discharged. This perceived reduction in the number of patients reviewed was further compounded by a lack of computer terminals, which was often reported as a rate-limiting step. One pharmacist mentioned the introduction of ‘computer ownership’ on the wards, whereby groups of staff would designate computers for their own use.

*“So I think first of all trying to get a computer is an issue. I work on one particular ward and they’ve basically put stickers on a lot of their computers-on-wheels saying ‘This is for nurses’ use’, which is really difficult for you as a pharmacist [……] you end up going and writing it all down on paper and then going back to a computer and sitting down and putting it all onto [the ePA system] and that’s really time consuming because you’re duplicating the work ….”* (Pharmacist 4; 6.5 years’ experience)

An important unintended consequence of ePA was that pharmacists perceived they were interacting with fewer patients, and that although the conversations held were more targeted (e.g. focused around a specific question), they were of a less natural mode.

*“I definitely think it [the ePA system] cuts down on patient interaction … So, yeah, unfortunately I think it definitely cuts down on patient interactions and meaningful patient interactions … Previously you would have been seeing everyone on a daily basis at their bedside but now if you can’t find a computer you don’t do that and more commonly the purpose for going is to get the drug history or to ask questions rather than going along on a day to day basis and checking in with the patient”.* (Pharmacist 4; 6.5 years’ experience)

*“Taking a device to the bedside still kind of takes away a little bit from a natural conversation because of the inputting you’re doing at the same time as having a conversation with someone[…….] And I feel like with a chart pre [ePA] implementation, the conversation was a little bit more natural”.* (Pharmacist 1; 4 years’ experience)

#### Perceived impact of ePA on patient safety

A prevalent belief was that ePA had increased the prevalence of medication errors (both minor and severe). Pharmacists also reported on examples of medication risks with ePA as compared with paper drug charts. Interview findings suggests the perceived increase in medication errors may be due to six types of situation: (i) ePA nudges users to do the ‘wrong’ thing, (ii) ePA indiscriminately defaults to specific actions, (iii) ePA makes error less visible compared to paper drug chart, (iv) ePA makes error more visible compared to paper drug chart, (v) ePA makes other key information less visible, and (vi) ePA clinical decision support does not meet users’ expectations. Examples are provided in Table 2. Five were risks that could be reduced by improving user training. Of these, four could be reduced with system improvement. The remaining factor was not a medication risk but a safety feature that raises awareness that an error has occurred, in that omitted doses were perceived to be more visible with ePA.

**Table 2:** Electronic prescribing and medication administration (ePA) features that contributed to perception of increased medication risk

ePA features	Implications for practice	Examples from pharmacist interviews
1. System nudges users to do the ‘wrong’ thing	These are risks that could be reduced with system improvements and/or better user training	“*So the doctors will often just click whichever one [insulin] is first, which is often, say, a cartridge of one and the patient’s got a FlexPen and it’s the pen that’s being used”* (Pharmacist 3; 2.9 years’ experience)
2. System indiscriminately defaults to specific actions		*“Like the doctors saying they’ve prescribed antibiotics, but if it’s past a certain timeframe it’ll automatically like set the next time. If you prescribed it at like 10 am and it is twice a day, it’ll set to 10 pm and they don’t always remember to put like a stat [once only] dose to be given straight away”* (Pharmacist 3; 2.9 years’ experience)
3. System makes error less visible (than a paper drug chart)		*“I see a lot more duplication of prescriptions than I did on paper drug charts. I think that doctors are not necessarily looking at the drug chart properly in the same way that you might with a paper drug chart. Duplication is something I see day in, day out; they’re asked to prescribe something and they don’t seem to realise the patient’s already on it”.* (Pharmacist 4, 6.5 years’ experience) *“So missed doses sometimes I think [on ePA] aren’t as apparent or there is a way that they can drop off … I think it [missed doses] was more apparent on a paper chart … So there are ways where you view the drug charts for a time period and you have to have your options and your settings… Your default setting at what you look at has to be like big enough or cover a time period that is big enough to show you things like that dose was missed yesterday. Because if you’re just looking at today’s drug administration then you won’t see that. So that’s kind of the system has created that. Whereas on a paper chart you would see kind of the prescription and it would show you all the days for that 14 days”. (Pharmacist 1, 4 years’ experience)*
4. System makes error more visible (than a paper drug chart)	This is a safety feature that raises awareness of an error	*“I think missed doses are more obvious now. Obviously on paper you didn’t have this constant reminder, whereas on [the ePA system] now, after an hour has elapsed from the time it was due, the box goes red, the administration box goes red.”* (Pharmacist 2; 1.5 years’ experience)
5. ePA makes other key information less visible (compared to paper drug charts)	This is a risk that could be reduced with system improvements and/or better user training	*“There’s also annotating some of the prescriptions as well for like therapeutic drug monitoring, which is probably a downside to [the ePA system] in some respects in that previously, for example, aminoglycosides, you would write ... gentamicin, the level less than 1, [then] you’d put a box as to when they need to take the level, you could tell them to take the level then give or take the level then hold; you don’t have the opportunity to do that on the drug chart or on the administration chart on the electronic prescribing the way you could on a paper drug chart”.* (Pharmacist 4; 6.5 years’ experience)
6. System clinical decision support not meeting users’ expectations	This is a risk that could be reduced with better user training	*“Doctors … they prescribe something after 8 am and it doesn’t flag then as being due until the next day.”* (Pharmacist 2; 1.5 years’ experience)

Pharmacists also reported a number of ePA-based patient safety improvements e.g. every prescription is always clear and complete with medication order details, name and bleep number of the prescriber. This facilitates investigation of medication errors as well as feedback to those involved. The ePA system also prevents medications from being prescribed if there is a documented allergy to the drug concerned in the patient’s record. However, pharmacists believed that it was human safety nets, namely doctors, nurses and pharmacists looking at medication orders, which allowed errors to be rectified before they can cause severe harm.

*“I'd like to think that, because there are lots of different teams looking at the prescriptions, not just one person, that errors will be identified, similar to if they were written incorrectly. So I don't think it’s any more or less dangerous than a paper chart…”* (Pharmacist 6, 5 years’ experience)

## Discussion

### Key findings

This study has revealed a number of effects of implementing ePA on ward pharmacists’ activities. Some routine tasks took longer while others reduced post-ePA, which corroborates previous research [15, 16]. Pharmacists perceived a reduction in time spent with patients and visitors, which was supported by the small but statistically significant reduction identified from our quantitative results. However, there was neither a change in the overall time spent at patients’ bedside, nor a significant increase in time spent in the ward office or nurses’ station where a large number of computers were located, a finding that was not anticipated. Pharmacists valued a number of safety features associated with ePA but also perceived an overall increase in medication risk. We attributed this to six types of situations. The majority could be improved with enhanced user training but four would also benefit from system redesign.

### Interpretation

Our findings suggest a key underlying change post-ePA was the enhanced and shifting role of the ward pharmacist. Pharmacists generally perceived an enhanced ability to review patients’ medications more comprehensively and efficiently (despite perceiving some tasks to take longer), a greater transcribing role in ordering new medications on behalf of some doctors and that they were seen as an ePA expert among ward staff.

The enhanced pharmacists’ role may be associated with the perceived increase in the number of medication errors from a more thorough medication review, and the increase in time spent reviewing inpatient medications. Previous studies suggest a mixed picture of positive and negative impact of ePA on patient safety with respect to medication use [7, 14]. Our study builds on these findings by identifying six types of situations that are potentially linked to ePA, and which increased the *perception* of error (Table 2). In considering these factors and in the context of implementation, our findings suggest the perceived impact of ePA may also be attributed to any recent experience of using paper drug charts by pharmacists and other ward staff. Specifically, ‘ePA makes error less visible compared to paper drug chart’, ‘ePA makes error more visible compared to paper drug chart’, and ‘ePA makes other key information less visible’. The apparent contradiction of the same ePA feature making an error less or more visible by different pharmacists may be influenced by the time period for which the drug administration record was being viewed. Unlike previous paper drug charts which allowed users to record and view drug administrations for up to 14 days, the ePA records all drug administrations for the whole duration of the inpatient stay but only a small number fit into a single screen for viewing at any one time. Users can scroll backwards and forwards in time to view other drug administrations. This suggests any actions taken to address this perceived risk should consider the contexts in which the ePA feature can be used. There appears to be an implicit expectation from pharmacists that ePA would provide the ‘same functionalities and more’ than a paper drug chart. However, in practice, paper drug charts afforded users a degree of flexibility where they were able to annotate as necessary. Thus paper drug charts were being routinely adapted to communicate an array of information at a glance that was no longer as visible using ePA. For example, pharmacists reported not being able to annotate a box with ePA to indicate when the next gentamicin level was due (Table 2) - this communication was routinely used by pharmacists to provide drug monitoring advice and a reminder for action to be taken by ward staff.

With respect to the ‘ePA expert’ role, interviewed pharmacists perceived that others expect them to have greater knowledge of how to use the ePA system. In practice, pharmacy staff at the study site received the same level and quantity of training (adapted for role-specific tasks) as doctors and other ward staff. Given that ePA was implemented relatively recently in our study, it is possible that the pharmacist’s role is still changing. The additional ePA expert role may dissipate as greater ePA knowledge and experience becomes embedded among core staff across all disciplines.

Patients play an important role in medication safety and can contribute to reducing medication error when health professionals involve patients in their care [27, 28]. A common concern among pharmacists was the perceived reduction in patient contact time, which resonates with findings from a previous study with medical staff [14]. Similar to an earlier English study [15], our study suggests that the nature of interactions with patients changed from having more routine and opportunistic discussions to more targeted discussion around specific medications, particularly on the acute admissions ward. This may explain why the overall time spent with patients changed little post-ePA i.e. fewer patients were seen but they were seen for longer periods of time post-epA. While the clinical significance of reduced patient contact is unclear, pharmacist interviews suggest that patients are still viewed as an integral part of the medication review process.

### Strengths and limitations

A strength of this study was the use of a planned explanatory mixed methods approach. This not only allowed us to triangulate our findings but also allowed us to gain a more in-depth understanding of contributing factors for post-ePA effects observed. In particular, the same pharmacists participated in both the quantitative and qualitative parts of the study, which ensured interviews were of maximum relevance to the observational data collected. Furthermore, there was a high level of engagement and involvement from pharmacy staff at multiple stages of the research (including study design, pilot work, data analysis and write-up).

Limitations include lack of data on tasks carried out by pharmacists outside of the ward visit, not observing the acute admissions pharmacist for the whole day, a small sample of observations, data being collected starting immediately post-implementation which will therefore include any ‘settling in’ time, the presence of an observer potentially affecting the way the pharmacists worked, and lack of data on the tasks carried out by other pharmacy staff who were supporting the observed pharmacists. Our study was also conducted at one hospital and therefore may not be generalizable to other settings. Finally, we used a relatively large number of different task types, compared to other studies, that provided us with greater granularity of potential specific effects of ePA, but it also reduced the statistical power for detecting differences.

### Implications for practice

It was recognised within the pharmacy department concerned that revision to existing guidance on clinical ward service provision was needed to better support individuals and teams to effectively prioritise and deliver high quality care post-ePA. Our findings support this and provide useful insights on how ePA has influenced pharmacists’ work processes on an acute admissions and a medicine-for–the-elderly ward. We suggest updating guidance on specific work processes (e.g. using ePA to document queries arising from clinical screening of inpatient medications, recording medication history and medicines reconciliation) to maximise the efficiency of having a centralised system for written communication, and supporting pharmacists to involve patients routinely when reviewing their medications using a mobile computing device on the ward. We also suggest actively encouraging staff to report any difficulties or workarounds with ePA to the pharmacy ePA team to facilitate shared learning and identity actions as required to address any problems. Furthermore, we recommend increasing the number of mobile computing devices available to ward pharmacy staff, and a review of the location of computer terminals on the wards to facilitate greater access to the ePA system.

### Future research

The perceived increase in medication errors is a concern and we suggest future research to develop and evaluate methods to monitor the extent to which ePA-related and non-ePA errors are captured in routine practice. We also suggest capturing types of remedial actions to facilitate organisational learning and greater shared learning across the health sector. Finally, we suggest further research should seek to map and better understand the unintended consequences of ePA, particularly on how it may enhance (or potentially generate more) work for health professionals in order to improve patient safety. Any system design should also consider how users may change in the short, medium, and long term as appropriate. For example, pharmacists who have never worked with paper charts - and consequently never experienced how patient contact naturally arises from bedside screening of paper charts [7] or how regular patient contact provides greater opportunities for a patient to contribute to increased medication safety [27] may benefit from a clear patient-focused practice model.

## Conclusion

This study provides quantitative and qualitative insights into the effects of implementing ePA on ward pharmacists’ activities. Some tasks took longer while others reduced, and our results suggest that pharmacists may spend less time with patients with ePA. Pharmacists valued a number of safety features associated with ePA but also perceived an overall increase in medication risk. Pharmacy staff demonstrated a degree of resilience to ensure ‘business as usual’ by enhancing and adapting their role.

## Additional files

Additional file 1: Quantitative work-sampling task definitions with inclusion and exclusion examples, adapted from Schofield et al. [23]. (PDF 59 kb)

Additional file 2: Topic guide for semi-structured interviews with hospital pharmacists. (PDF 492 kb)

Additional file 3: Percentage of time spent on tasks pre- and post-electronic prescribing and medication administration (ePA). (PDF 410 kb)

## Abbreviations

CPOE: computerised physician order entry
ePA: electronic prescribing and medication administration
NHS: National Health Service
