# Supplementary material for: The impact of implementing a hospital electronic prescribing and administration system on clinical pharmacists’ activities - a mixed methods study
Source: BMC Health Serv Res. 2019 Mar 12;19:156. doi: 10.1186/s12913-019-3986-4 (PMC6417214; doi:10.1186/s12913-019-3986-4)
Supplement: Supplementary file 1 — Quantitative work-sampling task definitions with inclusion and exclusion examples, adapted from Schofield et al. [23]. (PDF 59 kb) [file 12913_2019_3986_MOESM1_ESM.pdf]

Additional file 1 Quantitative work-sampling task definitions with inclusion and exclusion examples, adapted from Schofield et al [1]. Legend: Tasks are grouped into those that are value-added and non-value-added from a patient's perspective, based on Lean methodology, to facilitate identification of potentially 'wasteful' or non-value-added steps [2]. Codes listed were those used during data collection.

Code	Task	Task definition	Inclusions and exclusions
	**A. Value-added tasks**		
**RxI**	1. Prescribing medication (Inpatient)	This involves prescribing medication for inpatient administration.	Includes: Writing/entering a new medication order, changing orders, and giving a verbal order for inpatient administration<br>Excludes: Re-writing/re-entering orders (eg re-writing due to legibility issues, see **Transc**), prescribing for discharge (**RxD**)
**RxD**	2. Prescribing medication (Discharge)	This involves prescribing medication and/or completing discharge information to communicate plan for a patient's treatment at discharge.	Includes: Writing/entering a new medication order, changing orders, and giving a verbal order for discharge<br>Excludes: Re-writing/re-entering orders (eg re-writing due to legibility issues, see **Transc**), writing a new inpatient medication order, changing inpatient orders, giving verbal orders for inpatient administration **(RxI)**.
**Transc**	3. Transcribing medication orders	This involves transferring medication orders previously written for inpatient use	Includes: Copying medication orders from one medication chart/screen to a new medication chart/screen, re-writing orders following a request for clarification<br>Excludes: Writing/entering a new medication order, changing orders, and giving a verbal order (**RxI** for inpatient use, **RxD** for discharge)
**DHx**	4. Drug history	This involves taking a drug history from a patient and/or other people/materials. Taking a drug history is one part of the medicines reconciliation process. Other parts of the medicines reconciliation are recorded separately: screening inpatient medication, searching for information, reviewing patient records, and medication discussion.	Includes: Speaking with a patient/carer about their drug history, going through a patients' own drugs, contacting a health care professional to obtain a patient's drug history, documenting the drug history<br>Excludes: Discussing medications that are unrelated to the drug history with a patient/carer or health professional (**DisT** or **DisC**). Discussions with a patient/carer that are not medication-related (**Care**).
**DisT**	5. Medication discussion (technical)	This involves discussing any legal requirements, clarification and/or any other non-clinical aspects of a medication order for inpatient use or discharge	Includes: Asking a prescriber to re-write/re-enter a drug order due to illegibility or legal reasons, asking another health professional to clarify the drug order, clarifying with a patient/carer about the drug order, telling another healthcare professional about the location of a medication (not searching)<br>Excludes: Discussing a new medication order for inpatient administration or discharge (**DisC**), discussing drug history (**DHx**)
**DisC**	6. Medication discussion (clinical)	This involves discussing any clinical aspects of a medication order for inpatient use or discharge	Includes: Clarifying the choice of drug or dosage, discussing side effects, discussing administration protocols, discussing drug choices (dose, frequency, compliance) with patient/relative, asking doctors to prescribe/amend medication order<br>Excludes: Asking a prescriber to re-write/re-enter an illegible or incomplete order (**DisT**), discussing drug history (**DHx**)
**H**	7. Handover	This involves reviewing a handover or documenting on a handover sheet or electronic handover	Includes: Documenting on handover, reviewing (viewing) handover, printing handover<br>Excludes: Discussing a patient's drugs using the handover **(DisC)**, non medication-related communication **(Comm)**
**Order**	8. Ordering medication	This involves placing/cancelling/chasing a request for mediation supply	Includes: Making or taking a request (verbal, telephone, faxing) for medication supply, chasing medication to be supplied<br>Excludes: Ordering medication from a discharge prescription **(ScrD)**
**Disp**	9. Dispensing medication	This involves labeling, retrieving, dispensing and/or checking medications for supply against a medication order	Includes: Amending an existing label on a medication container, searching for medication, asking for a check of the medication supplied, checking the medication supplied is the same as on the medication order, clarifying medication order for dispensing<br>Excludes: Ordering a medication (**Order**), giving out medication to a healthcare professional/patient/carer (**O**), telling a healthcare professional the location of the medication supplied (**DisT**)
**ScrI**	10. Screening Inpatient medication	This involves clinically screen drug orders as part of planning and reviewing inpatient medication	Includes: Reviewing a patient's medication for appropriateness, adding instructions to medication orders, endorsing medication orders with drug supply information for inpatient use

Code	Task	Task definition	Inclusions and exclusions
		treatment	Excludes: reviewing medications for discharge (**ScrD**), discussing medication (**DisT** or **DisC**), drug history taking (**DHx**)
**ScrD**	11. Screening discharge medication	This involves clinically screen and add instructions to a patient's discharge summary	Includes: Adding instructions to medication orders, endorsing medication orders with drug supply information for discharge, adding or printing information to discharge summary letter<br>Excludes: Reviewing medications for inpatient use (**ScrI**), discussing medication (**DisT** or **DisC**), drug history taking (**DHx**)
**Care**	12. Helping patient/carer (non-medication related)	This involves any activity that is perceived as helping a patient by the observer.	Includes: Escorting a patient, communicating with patient/carer or visitor, making a patient comfortable, transporting a patient. All non-medication related communication with patient/carer is defined as direct care.<br>Excludes: Medication related activities, communicating with staff member or patient regarding medications (**DisT** or **DisC**).
**Comm**	13. Professional communication (not medication)	This involves any work-related discussion with another staff member that is not medication related	Includes: Requesting medical or nurse review, planning care with any health professional, handover/parts of a ward round, telephone call<br>Excludes: Medication related discussion with staff member or patient/carer (**DisT** or **DisC** or **DHx**)
**RevR**	14. Reviewing patient records	This involves the review and/or documentation of information held on a patient's paper or electronic medical record	Includes: Looking up laboratory results, patient's bedside observation records, medical notes, writing in a patient's medical notes<br>Excludes: Reviewing the medication chart/screen (**ScrI** or **ScrD**), reviewing a patient's list of medications from home (**DHx**)
**B.**	**Non-value-added tasks**		
**SearM**	15. Searching for paper drug chart or computer	This involves searching for a medication chart/device to access medication orders that was not found in its usual location	Includes: Looking for medication charts with drug order, asking about the location of the paper/electronic medication order, looking for a tablet device to access the electronic medication order<br>Excludes: Looking for a patient's medical notes, looking for equipment, waiting to access the medication order if someone else is using it (**WaitM**)
**SearI**	16. Searching for information	This involves searching for information on different drug treatments using the computer or other resources	Includes: Searching information using the computer or paper-based reference resources<br>Excludes: Searching for medical notes (**O**) , asking another healthcare professional (**DisT**)
**WaitM**	17. Waiting for paper drug chart/access to medication order	This involves time spent waiting to access a drug chart/electronic mediation order	Includes: Waiting for medication charts and/or tablet device to access the electronic medication order, waiting for computer device to load medication order screen<br>Excludes: Looking for a patient's medical notes (**O**), looking for equipment (**SearM**)
**Trav**	18. Travelling	This involves time spent walking between patients and tasks.	Includes: Walking to answer a phone or bleep, walking to a new task<br>Excludes: Travel associated with any of the pre-defined tasks in this table (**Trav**)
**Edu**	19. Supervising a pre-registration pharmacist	This involves the time spent on supervising and educating a pre-registration pharmacist	Includes: Showing the pre-registration pharmacist how to perform a task or explaining to the student the reason behind a specific treatment
**C.**	**Other**		
**O**	20. Other	Any task that is not included in the list of pre-defined tasks in this table.	

References

1. Schofield B, Cresswell K, Westbrook J, Slee A, Girling A, Shah S, et al. The impact of electronic prescribing systems on pharmacists' time and workflow: protocol for a time-and-motion study in English NHS hospitals: Table 1. BMJ Open. 2015;5:e008785. doi:10.1136/bmjopen-2015-008785.

2. Jones D, Mitchell A, Lean Enterprise Academy UK. Lean thinking for the NHS. London; 2006.
