# Supplementary material for: The impact of implementing a hospital electronic prescribing and administration system on clinical pharmacists’ activities - a mixed methods study
Source: BMC Health Serv Res. 2019 Mar 12;19:156. doi: 10.1186/s12913-019-3986-4 (PMC6417214; doi:10.1186/s12913-019-3986-4)
Supplement: Supplementary file 2 — Topic guide for semi-structured interviews with hospital pharmacists. (PDF 492 kb) [file 12913_2019_3986_MOESM2_ESM.pdf]

Additional file 2. Topic guide for semi-structured interviews with hospital pharmacists. The name of the electronic prescribing and medication administration (ePA) has been replaced with 'ePA' in the present version.

<u>Introduction</u>:

- Hello, good morning / afternoon.
- Thank you very much for agreeing to take part in this interview.
- We are trying to investigate what are the perceptions and the thoughts of pharmacists regarding the impact that ePA (and particularly the inpatient electronic prescribing and administration system) has on their ward, medication-related activities. There are no right or wrong answers.
- I really appreciate the time you will invest for this interview.
- With your permission, can I record the interview?

<u>Questions:</u>

This is the beginning of interview No…………………….

- With your permission, which ward do you usually cover?
- With your permission, how many years of experience do you have working as a pharmacist?

1. What do you think about the ePA system for carrying out your daily ward activities?
Prompts:

- What would you say are the benefits and disadvantages of the ePA system for you when carrying out ward activities and why?
- Could you tell me a bit more about that?
- Can you give me an example of that?

2. Could you describe how ePA has affected your work?
Prompts:

- Are there any activities that you no longer need to do (compared with the old paper drug chart system) and why?
- What is the impact on the time you need to carry out your ward activities and why?
- How it affects the way you approach to your ward activities?
- Could you tell me a bit more about that?
- Can you give me an example of that?

3. What do you think is the effect of ePA on medication errors (any kind of errors such as prescribing, administration, dispensing and monitoring errors)?
Prompts:

- Can you describe any medication errors specific to ePA?
- Are there any medication errors no longer happening after the implementation of ePA?
- What is the impact on the severity of medication errors?
- What is the overall impact on the frequency of medication errors?
- Can you give me an example of that?

- You mentioned …………… Would you tell me more about this?

4. What do you think is the impact of the ePA on interactions you have with patients?
Prompts:
- Has it changed the way you interact with patients (either the mode or the purpose of interaction) and why?
- Has it changed the amount of time you spend with patients and why?
- Can you describe any new type of interactions with patients emerged after the implementation?
- Are there any interactions with patients no longer taking place (compared with when drug charts were placed next to the beds)?
- Do you prefer the system or the patient as a source of reliable information?
- Can you explain that a bit more?
- Can you be a bit more specific about that?

5. What do you think is the impact of ePA on interactions you have with other health-care professionals?
Prompts:
- Has it changed the way you interact with other health-care professionals (either the mode or the purpose of interaction) and why?
- Has it changed the amount of time you spend with other health-care professionals and why?
- Can you describe any new type of interactions with other health-care professionals emerged after the implementation?
- Are there any interactions with other health-care professionals no longer taking place (compared with the old paper drug chart system)?
- Can you explain that a bit more?
- Can you be a bit more specific about that?

6. Could you describe how ePA affects the locations in which you carry out your ward activities?
Prompts:
- Can you describe any new locations that you now carry out your ward activities?
- Are there any locations that you no longer need to carry out ward activities?
- Can you give me more details about that?
- Can you give me an example of that?

Conclusion: Thank you very much indeed for taking part in this interview. Is there anything else you have in your mind that you would like to mention? Do you have any questions you would like to ask? Is there anything else you would like me to explain?
Again thank you very much for your time.
Have a great day.
