# Supplementary material for: The impact of implementing a hospital electronic prescribing and administration system on clinical pharmacists’ activities - a mixed methods study
Source: BMC Health Serv Res. 2019 Mar 12;19:156. doi: 10.1186/s12913-019-3986-4 (PMC6417214; doi:10.1186/s12913-019-3986-4)
Supplement: Supplementary file 3 — Percentage of time spent on tasks pre- and post-electronic prescribing and medication administration (ePA). (PDF 410 kb) [file 12913_2019_3986_MOESM3_ESM.pdf]

Additional file 3. Percentage of time spent on tasks pre- and post-electronic prescribing and medication administration (ePA).

**Task (in descending order of estimated time pre-ePA)**	**Pre-ePA**			**Post-ePA**			**p-value* (unpaired t-test)**
	Activity samples	Percentage of total time	Estimated time overall (h:min)	Activity samples	Percentage of total time	Estimated time overall (h:min)	
Professional communication (not medication)	205	15.9	6:56	329	15.1	11:05	0.62
Screening inpatient medication	149	11.6	5:02	378	17.4	12:44	0.20
Screening discharge medication	142	11.0	4:48	135	6.2	4:33	0.45
Medication discussion (clinical)	110	8.6	3:43	156	7.2	5:15	0.37
Reviewing patient records	107	8.3	3:37	172	7.9	5:48	0.91
Drug history	83	6.5	2:48	287	13.2	9:40	0.02
Travelling	77	6.0	2:36	113	5.2	3:49	0.96
Handover	69	5.4	2:20	148	6.8	4:59	0.37
Ordering medication	55	4.3	1:52	84	3.9	2:50	0.75
Supervising a pre-registration pharmacist	51	4.0	1:43	13	0.6	0:26	0.76
Dispensing medication	40	3.1	1:21	30	1.4	1:06	0.95
Medication discussion (technical)	35	2.7	1:11	62	2.8	2:05	0.62
Searching for information	18	1.4	0:37	64	2.9	2:09	0.30
Waiting for paper drug chart/access to medication order	18	1.4	0:37	61	2.8	2:03	0.07
Helping patient/carer (non-medication related)	18	1.4	0:37	14	0.6	0:28	0.50
Transcribing medication orders	16	1.2	0:32	40	1.8	1:21	0.11
Searching for paper drug charts or computer	15	1.2	0:30	0	0	0:00	0.00
Prescribing medication (inpatient)	0	0.0	0:00	0	0	0:00	---
Prescribing medication (discharge)	0	0.0	0:00	0	0	0:00	---
Other	79	6.1	2:40	90	4.1	3:02	0.49
Total	1287	100	43:49**	2176	100	73:26**	

*statistical significance based on Bonferroni p-value <0.0028

**Total estimated time does not match total observed times of 43 hours 30 mins pre-ePA and 73 hours 33 mins post-ePA due to rounding of numbers.
